# Preparation and evaluation of high-performance modified poly(ester-amide), and study its biological activity after its formulated as a vehicle for protective coating

**DOI:** 10.1038/s41598-026-62902-8

**Published:** 2026-07-21

**Authors:** Hamada Abd El-Wahab, Mahmoud M. Fayad, Mahmoud H. Hendy, Salem S. Salem, Ammar M. Mahmoud

**Affiliations:** 1 Chemistry Department, Faculty of Science (Boys), Al-Azhar University, Nasr City, Cairo Egypt; 2 Egyptian Petroleum Research Institute, Nasr City, Cairo 11727 Egypt; 3 Botany and Microbiology Department, Faculty of Science, Al-Azhar University, Nasr City, Cairo 11884 Egypt

**Keywords:** Modified polyester resins, Paint formulation, Mechanical properties, Biological activities, Chemistry, Materials science, Microbiology

## Abstract

Poly(ester-amide) (PEA) resins are widely used in coating applications because of their excellent mechanical strength, chemical resistance, and durability; however, the development of PEA coatings with enhanced antimicrobial performance remains a significant challenge. In this study, a novel modified PEA resin was synthesized using 3-*N*-bis(2-hydroxyethyl)-7-hydroxy coumarin carboxamide (HEHCCA) as a reactive modifier. To the best of our knowledge, the incorporation of HEHCCA into PEA coating systems has not been previously reported. The modified resins were prepared by partially replacing hydroxyethyl linseed oil fatty acid amide (HELA) with HEHCCA, while phthalic anhydride was used as the polyacid component. The prepared resins were formulated into coating systems and characterized according to standard ASTM methods to evaluate their physicochemical, mechanical, chemical, and antimicrobial properties. The results showed that increasing HEHCCA content increased the reaction time, viscosity, color intensity, and drying time of the coatings. Mechanical properties such as gloss, impact resistance, scratch hardness, and adhesion improved noticeably, whereas flexibility remained nearly unchanged. The coatings also exhibited good chemical resistance except under alkaline conditions. In addition, the modified PEA resins demonstrated significant antimicrobial activity, particularly against *Staphylococcus aureus* and *Klebsiella pneumoniae*, with inhibition zones reaching 24.15 ± 0.30 mm. These findings suggest that HEHCCA-modified PEA resins are promising candidates for advanced protective and antimicrobial coating applications.

## Introduction

Poly(ester-amide) (PEA) resins represent an important class of polymeric materials that combine the advantageous characteristics of both polyesters and polyamides within the same macromolecular structure. The coexistence of ester (–COO–) and amide (–CONH–) linkages imparts a unique balance of flexibility, toughness, adhesion strength, thermal stability, chemical resistance, and film-forming ability, making PEA resins highly attractive for coating, biomedical, and advanced material applications. Compared with conventional alkyd and polyester resins, PEA systems exhibit superior mechanical durability and enhanced resistance toward solvents, corrosion, and environmental degradation due to the presence of polar amide groups capable of intermolecular hydrogen bonding^1–4^. Polymeric systems based on PEA resins have been extensively investigated as surface-coating binders because of their superior film-forming characteristics, antimicrobial activity and corrosion resistance properties^5–7^. In comparison with conventional alkyd resins, vegetable-oil-based PEA resins exhibit enhanced drying behavior, hardness, water resistance, and thermal and chemical stability. Moreover, the incorporation of functional modifiers and nanomaterials into PEA matrices has been shown to significantly improve coating performance and biological activity^8,9^. Several studies have reported the development of modified PEA systems using renewable vegetable oils such as Jatropha curcas, soybean, linseed, Moringa oleifera oils and other different oils^10–13^. Boron-modified polyesteramide systems also demonstrated enhanced antibacterial and antifungal activities^14^. Previous studies also demonstrated that modified PEA resins containing aromatic amide structures and free amino groups possess significant antimicrobial activity against both Gram-positive and Gram-negative bacteria, including *Escherichia coli* and *Staphylococcus aureus*^15,16^. Recently, coumarin-containing thiazole derivatives have been incorporated into polyurethane coatings to improve their antimicrobial performance without adversely affecting the physical and mechanical properties of the coating films^17,18^. In recent years, considerable research attention has been directed toward the development of modified PEA resins for high-performance surface coatings. The increasing demand for environmentally stable, mechanically durable, and biologically active coatings has encouraged researchers to incorporate functional organic moieties into PEA backbones to improve their physicochemical and antimicrobial properties. Functional modification can significantly influence resin viscosity, drying behavior, gloss, hardness, adhesion, flexibility, and chemical resistance through alterations in crosslink density and intermolecular interactions. Such modifications also provide opportunities for introducing bioactive functionalities into coating matrices^19–22^. Among the various functional compounds used for polymer modification, coumarin derivatives have attracted particular interest because of their remarkable biological and photochemical properties. Coumarin-containing compounds exhibit broad-spectrum antimicrobial, antifungal, antioxidant, anti-inflammatory, and ultraviolet-absorbing activities. The incorporation of coumarin moieties into polymeric coatings can therefore provide multifunctional characteristics, including enhanced antimicrobial performance and improved coating durability. Furthermore, hydroxyl-containing coumarin derivatives can actively participate in polyesterification reactions, allowing their incorporation into the polymer backbone as reactive modifiers rather than simple additives, which improves compatibility and long-term stability of the resulting coatings, The incorporation of coumarin moieties into polymeric coatings can provide multifunctional characteristics, including enhanced antimicrobial activity, photo-responsiveness, self-healing behavior, and improved coating durability owing to the unique photochemical and biological properties of coumarin derivatives^23–26^. Recently, antimicrobial polymer coatings have become increasingly important because microbial contamination and biofilm formation on surfaces present major challenges in healthcare, food packaging, marine systems, and industrial applications. Advanced antimicrobial coatings are designed to inhibit microbial adhesion and suppress the growth of bacteria and fungi on coated surfaces. Polymeric coatings containing biologically active heterocyclic or aromatic compounds have shown promising effectiveness due to their ability to disrupt microbial cell membranes and interfere with cellular metabolism. In this context, modified PEA resins are considered promising candidates because their structural versatility enables the incorporation of various antimicrobial functionalities while maintaining desirable coating performance^27–30^. Several studies have reported the preparation of antimicrobial alkyd and PEA coatings modified with Schiff bases, phenolic compounds, quaternary ammonium salts, and other bioactive materials to enhance biological activity and surface protection. However, studies concerning the incorporation of 3-*N*-bis(2-hydroxyethyl)-7-hydroxy coumarin carboxamide (HEHCCA) into PEA resin systems remain very limited. The presence of hydroxyl and amide functional groups in HEHCCA makes it highly suitable for chemical incorporation into polyester-amide networks, potentially enhancing intermolecular hydrogen bonding, crosslink density, coating adhesion, and antimicrobial activity simultaneously. Therefore, the present study focuses on the synthesis and characterization of modified PEA resins based on the partial replacement of hydroxyethyl linseed fatty acid amide (HELA) with HEHCCA as a reactive modifier. The newly prepared resins were evaluated in terms of their physicochemical characteristics, mechanical performance, chemical resistance, and antimicrobial activity. The incorporation of HEHCCA into the PEA structure is expected to produce multifunctional coating materials with improved durability and enhanced antimicrobial efficiency suitable for advanced protective coating applications.

## Experimental

### Materials

All chemicals used during this study were obtained from local markets or global firms. They were all pure and used without further purification. These materials are: Phthalic anhydride (PA), purity typically 99.5% and diethanolamine (DEA), 99% as supplied by SD Fine-Chem Ltd, Indian. Linseed oil fatty acid (LOFA) purity is 98% and supplied from Eagle Company, Egypt. Xylene and mineral turpentine products were obtained from Misr Petroleum Company, Egypt. Glycerol and phenolphthalein 99% as supplied by the El-goumhouria Co. BYK 8761 and BYKANOL-A (BYK-Chemie GmbH): “BYK 8761 (wetting and dispersing agent) and BYKANOL-A (anti-skinning agent) were obtained from BYK-Chemie GmbH (Germany)”.

### Methods and techniques

7-Hydroxy-3-carboxycoumarin was prepared through a one-pot consecutive reaction in aqueous medium using 2,4-dihydroxybenzaldehyde and malononitrile as starting materials. In the first stage, a Knoevenagel condensation reaction occurred between the aldehyde group of 2,4-dihydroxybenzaldehyde and malononitrile to form an intermediate imine derivative. Subsequently, the intermediate underwent in situ hydrolysis via a Pinner-type reaction, leading to the formation of 7-hydroxy-3-carboxycoumarin as the final product^31^.

#### Syntheses of 3-*N*-bis-(2-hydroxyethyl) 7-hydroxy coumarin carboxamide (HEHCCA), as a modifier

**Scheme 1:**
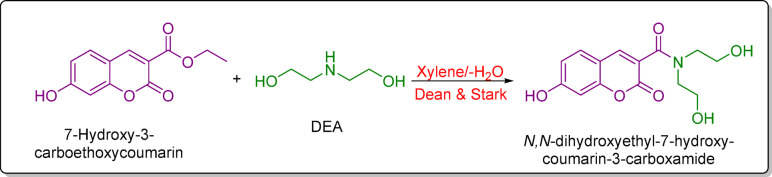
Preparation of -N-Bis-(2-hydroxyethyl) 7-hydroxy coumarin carboxamide (HEHCCA), as a modifier.

The synthesis of 3-*N*-bis-(2-hydroxyethyl)-7-hydroxy coumarin carboxamide (HEHCCA) as a reactive modifier was carried out through the condensation reaction of 7-hydroxy-3-carboxycoumarin with freshly distilled diethanolamine. A mixture containing 0.1 mol of 7-hydroxy-3-carboxycoumarin and 0.1 mol of diethanolamine was heated under reflux for approximately 6 h in the presence of ZnO as a catalyst. After completion of the reaction, the obtained product was isolated and identified as 3-*N*-bis-(2-hydroxyethyl)-7-hydroxy coumarin carboxamide (HEHCCA), as illustrated in Scheme 1. The prepared compound exhibited a melting point of 90–91 °C, which is in good agreement with the reported literature values^7,15^.

#### Synthesis of modified poly(ester-amide) (PEA) resins

The synthesis was conducted in two stages. First, the N, N-bis(2-hydroxyethyl) linseed oil fatty acid amide (HELA) precursor was prepared by reacting freshly distilled diethanolamine (DEA) (10.5 g, 0.11 mol) with linseed oil fatty acid (LOFA) (28 g, 0.1 mol) in a flask equipped with a Dean-Stark apparatus. The mixture was refluxed at 150 °C in xylene (15 mL) until the theoretical amount of water (1.8 mL, 0.1 mol) was collected.

**Scheme 2:**
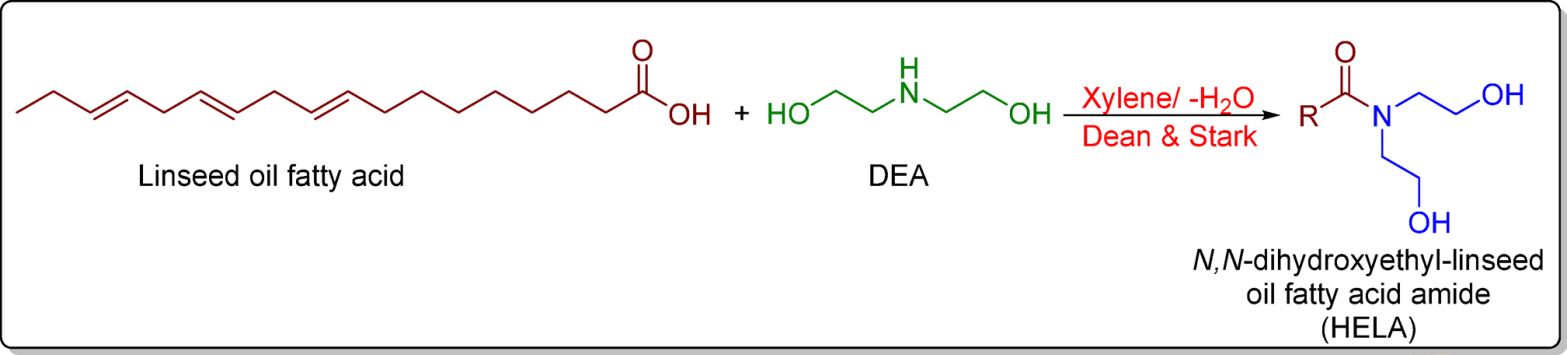
Synthesis of hydroxy ethyl linseed oil fatty acid amide (HELA).

**Scheme 3:**
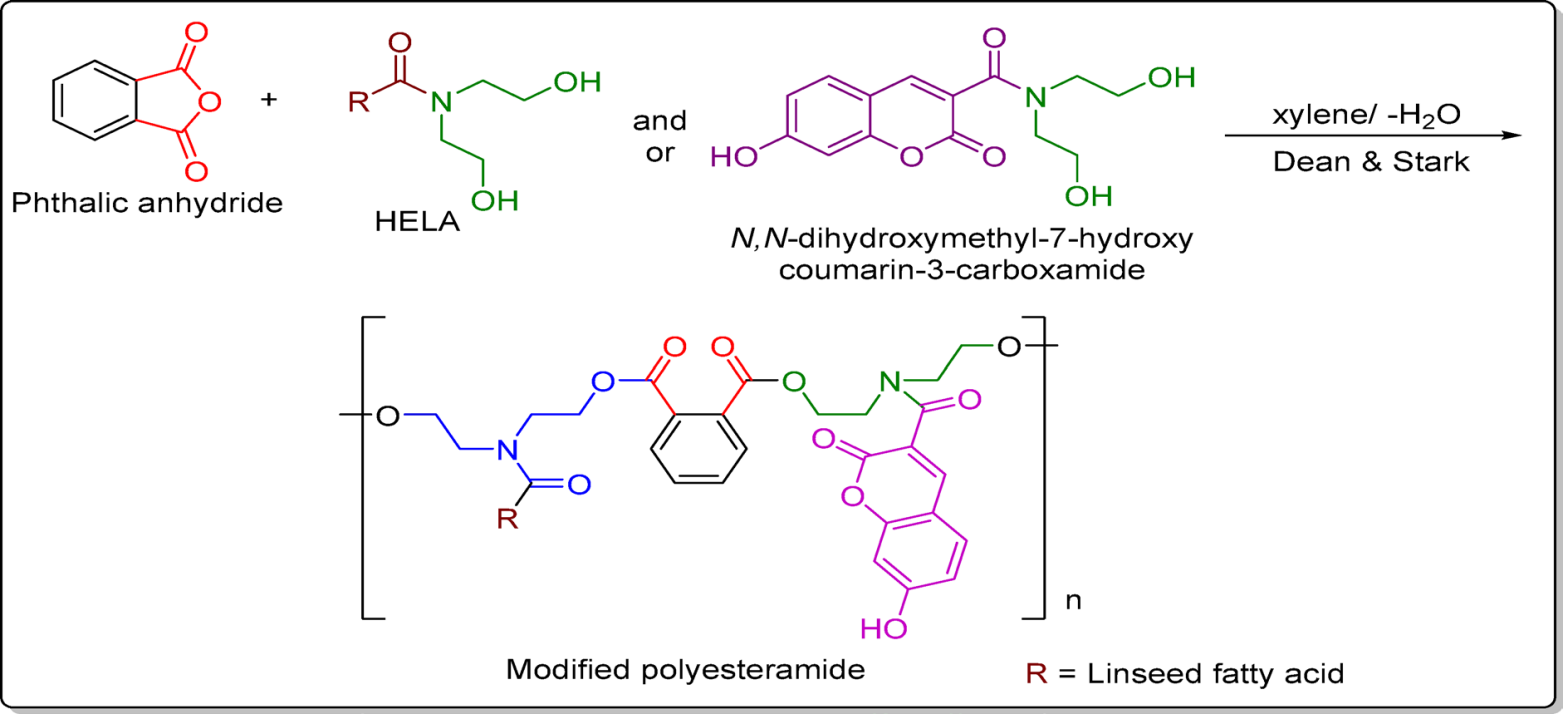
HEHCCA- modified poly(ester-amide) resins.

In the second stage, the modified PEA resins were formed by reacting phthalic anhydride (PA) as the dibasic acid components with HELA and the (HEHCCA) modifier as polyol source. The reaction was performed at 150 °C using 10% xylene as an azeotropic solvent. The esterification progress was monitored by the volume of water liberated according to the resin constants which represented in Table 1. Upon completion, the resins were blended with a specific combination of driers. The chemical structures of the prepared modified poly(ester-amide) resin are illustrated in Scheme 2 and 3^7,15^, .

**Table 1:** Resin constants of modified poly(ester-amide) (PEA).

No.ID	Ingredients	eo	E	eA	eB	F	mo= eo/F	*R*= eB/ eA	K= mo/eA	W= E. eo	H2O off ml
A	HELA (1.00) HECCA (0.00) PA	0.260 0.259	184 74.1	0.259	0.260	2 2	0.130 0.130	1	1	47.8 19.20	2.3
					0.260		0.260				
B	HELA (0.90) HECCA (0.10) PA	0.234 0.026 0.259	184 112 74.1	– – 0.259	0.234 0.026 –	2 2 2	0.117 0.013 0.130	1	1	43.10 2.912 19.20	2.3
				0.259	0.260		0.260				
C	HELA (0.80) HECCA(0.20) PA	0.208 0.052 0.259	184 112 74.1	– – 0.259	0.208 0.052 –	2 2 2	0.104 0.026 0.130	1	1	38.30 5.824 19.20	2.3
				0.259	0.260		0.260				
D	HELA (0.70) HECCA (0.30) PA	0.182 0.078 0.259	184 112 74.1	– – 0.259	0.182 0.078 –	2 2 2	0.091 0.039 0.130	1	1	33.48 8.736 19.20	2.3
				0.259	0.260		0.260				

**NB:-** **HELA** : Hydroxy Ethyl Linseed Amide, **PA** : Phthalic Anhydride, **HEHCCA** : Coumarin-3-*N*-bis-(2-hydroxyethyl) carboxamide, **E** : Equivalent Weight, **e**_**A**_: Number of acid equivalent, **e**_**B**_ : Number of hydroxyl equivalent, **e**_**0**_ : Total equivalent present at the start of the reaction, **F** : Functionality, **K** : Alkyl constant (m_0_ / e_A_), **R** : Ratio of total-OH groups to total-COOH groups (e_B_/ e_A_).

#### Coating formulation

The prepared modified poly(ester-amide) resins were incorporated into paint formulations to create antimicrobial coating compositions, as detailed in Table 2. These formulations were applied to mild steel panels using a brush method to achieve a dry film thickness of 105 ± 5 μm. Following application, the panels were air-dried for seven days at a temperature of 35 ± 2 °C and a relative humidity of 60 ± 5%. This period ensured complete drying, curing, and proper film adhesion to the substrate before testing. All subsequent tests were performed after this curing period.

**Table 2:** Semi-gloss paint formulation based on modified PEA resins.

Serial	Component	%
1	Resin / modified PEA resin	37
2	Bentone38	1.0
3	Ethanol	0.3
4	Wetting & dispersing agent (BYK 8761)	0.3
5	TiO_2_	10.0
6	Zinc phosphate	5.0
7	CaCO_3_	17.0
8	Talc	5.0
9	Xylene	22.21
10	Calcium octoate 10%	1.0
11	Cobalt octoate (10%)	0.43
12	Zirconium Octoate (12%)	0.63
13	Anti-skinning agent (BYKANOL-A)	0.13
Total		100

### Analytical techniques

IR spectra of the prepared compounds were obtained with a JASCO FTIR-4100. FT-IR spectrometer (Japan) operating in absorption mode in the wave number range of 4000–400 cm^− 1^ by the prepared compounds mixing with KBr (potassium bromide) discs.

^1^**HNMR spectra (DMSO-d6)** were obtained on JEOL (Japan Electron Optics Laboratory), chemical shifts were measured in δ ppm, relative to TMS as an internal standard (= 0 ppm), in the presence of DMSO as a solvent. The sample (3 wt%) was dissolved in DMSO with the internal standard tetramethylsilane.

### Characterization

#### Physical and mechanical properties of coatings

The following tests were carried out according to international standards: Test method for color of transparent liquid, ASTM method, D1544–04 (Reapproved 2010). Test method of viscosity, ASTM method, D4287–00 (Reapproved 2014). Steel substrates were prepared in compliance with ASTM D609-17 standards. Coating thickness was measured following ASTM D7091 using an Elcometer (Models 456 and 124) digital gauge. Hardness was assessed using a pencil hardness tester (ASTM D3363-11), while surface gloss was measured in accordance with ASTM D523-18 (TQC Sheen Glossmeter). Flexibility was evaluated using ASTM D522-17, and adhesion strength was tested with a crosshatch adhesion kit following ASTM D3359-17. Impact resistance, reflecting the coating’s ability to withstand rapid deformation, was determined using ASTM D2794-23, (Pendulum Impact Tester (GT-7045-HM). Water resistance of dried films, ASTM method, D870–15 Alkali resistance of dried films, Indian Standard Specification, 158(1950) Acid resistance of dried films, Indian Standard Specification, 159(1950) Solvent resistance of dried films, ASTM method, D2792-69 (Reapproved 2015).

#### Thermal analysis

A Shimadzu TGA-50 thermogravimetric analyser (Columbia, EUA) was used for the thermal analysis. Using a nitrogen environment, the samples were heated at a rate of 10 °C between ambient temperature and 600 °C.

#### Antimicrobial screening

The agar well diffusion method was used to assess Paint’s antimicrobial activity against *Escherichia coli* ATCC 8739, *Klebsiella pneumonia* ATCC 2146, *Bacillus subtilis* ATCC 6051, *Staphylococcus aureus* ATCC 25,923, and *Candida albicans* ATCC 90,028. In sterile Petri dishes filled with Mueller-Hinton agar, each bacterial strain that was cultivated in Mueller-Hinton broth was equally distributed. Using a sterile cork borer, 0.1 mL was added to 7 mm wells to test the paint’s antibacterial effectiveness. The inhibition zones were measured following a 24-h incubation period at 37 °C^32,33^. The negative control was a DMSO solution.

## Results and discussion

### Characterization of **spectral analysis**

**Fig. 1:**
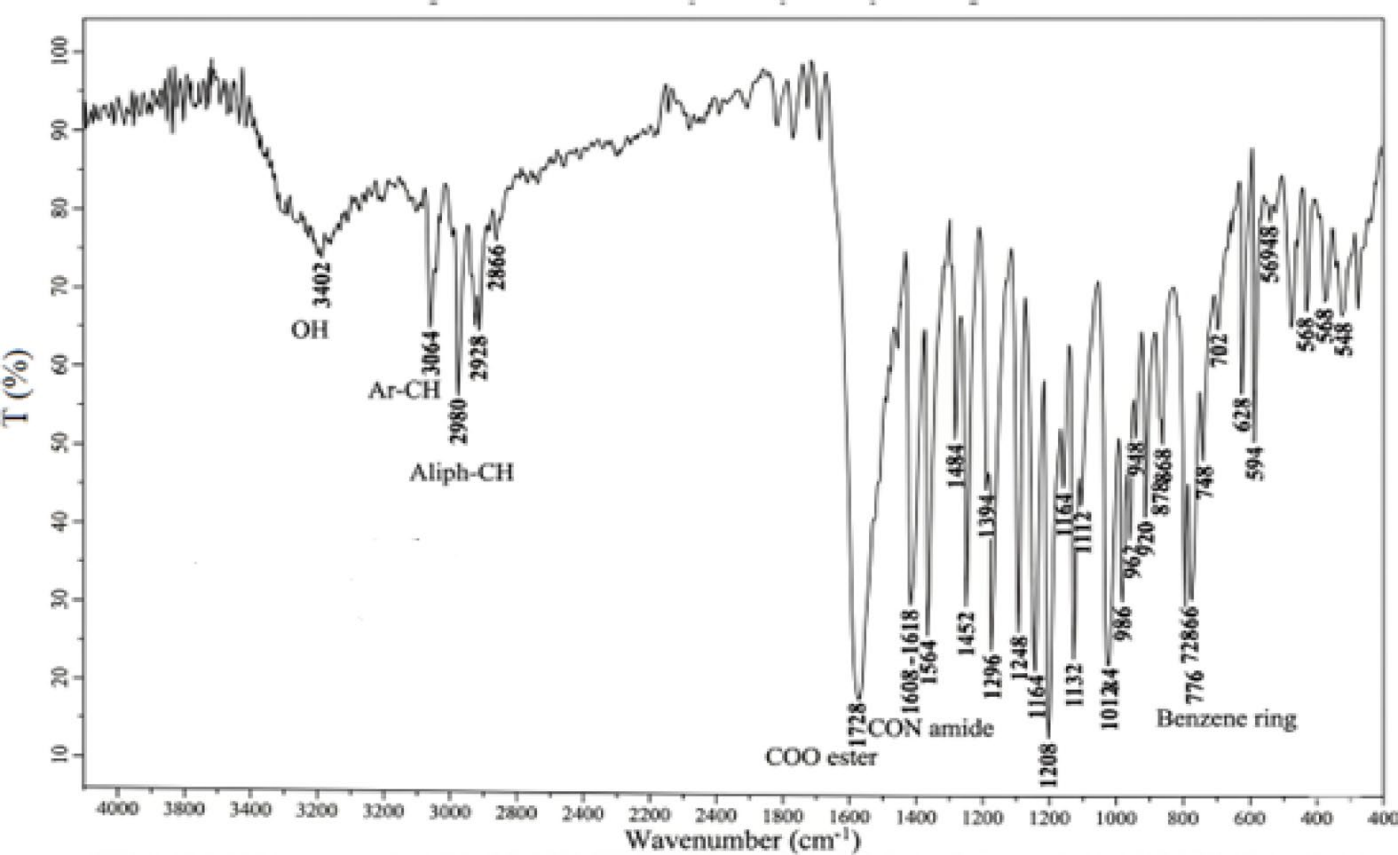
I.R Spectra of. N-N, bis (2-hydroxyethyle) 7, hydroxy coumarine carboxamide HEHCCA.

^1^***H-NMR spectrum*** (Fig. 2) was measured in DMSO-d^6^, and showed a coumarin ring as multiple signal at δ = 6.8–7.9 ppm. The peaks of CH_2_ attached to amide carbonyl (> N–(C=O)–CH_2_) at δ 4.7 ppm, and amide nitrogen ((–CH_2_)_2_ N–C=O) at δ 3.8 ppm. The peaks for protons of the alcoholic OH protons appeared at δ = 3.1 ppm. The peaks for protons of the phenolic OH protons appeared at δ = 5.0 ppm.

3-*N*-bis-(2-hydroxyethyl) 7-hydroxy coumarin carboxamide. Structural features associated with HEHCCA were confirmed by FT-IR analysis as shown in Fig. 1.

***I.R spectrum*** (Fig. 1), showed a broad band at about 3450 cm^− I^ which is characteristic for ν-OH. and band at about 2930–3070 cm^− I^ characteristic for aliphatic and aromatic CH group respectively, at 1778 cm^− 1^ is characterized for COO ester, at 1618 cm^− I^ is due to CON amide, the ν-CO. O in six membered ring at 1208 cm^− I^ and 1164 cm^− I^ and at about 776 cm^− 1^ due to disubstituted benzene ring^9,30^.

**Fig. 2:**
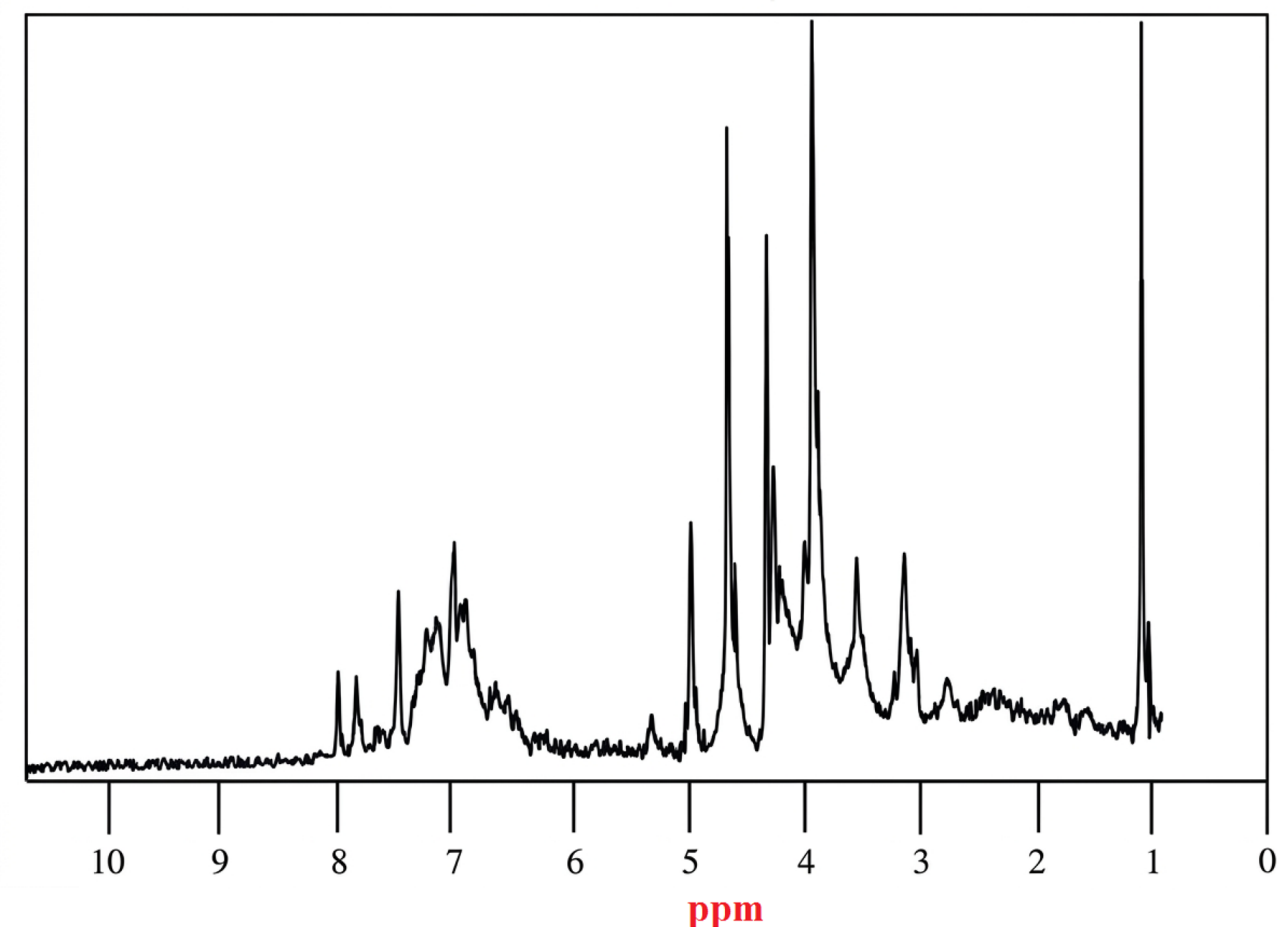
^1^H-NMR Spectra of. N-N, bis (2-hydroxyethyle) 7, hydroxy coumarin carboxamide (HEHCCA).

### Spectral analysis of HEHCCA- modified poly(ester-amide) resin (PEA)

**Fig. 3:**
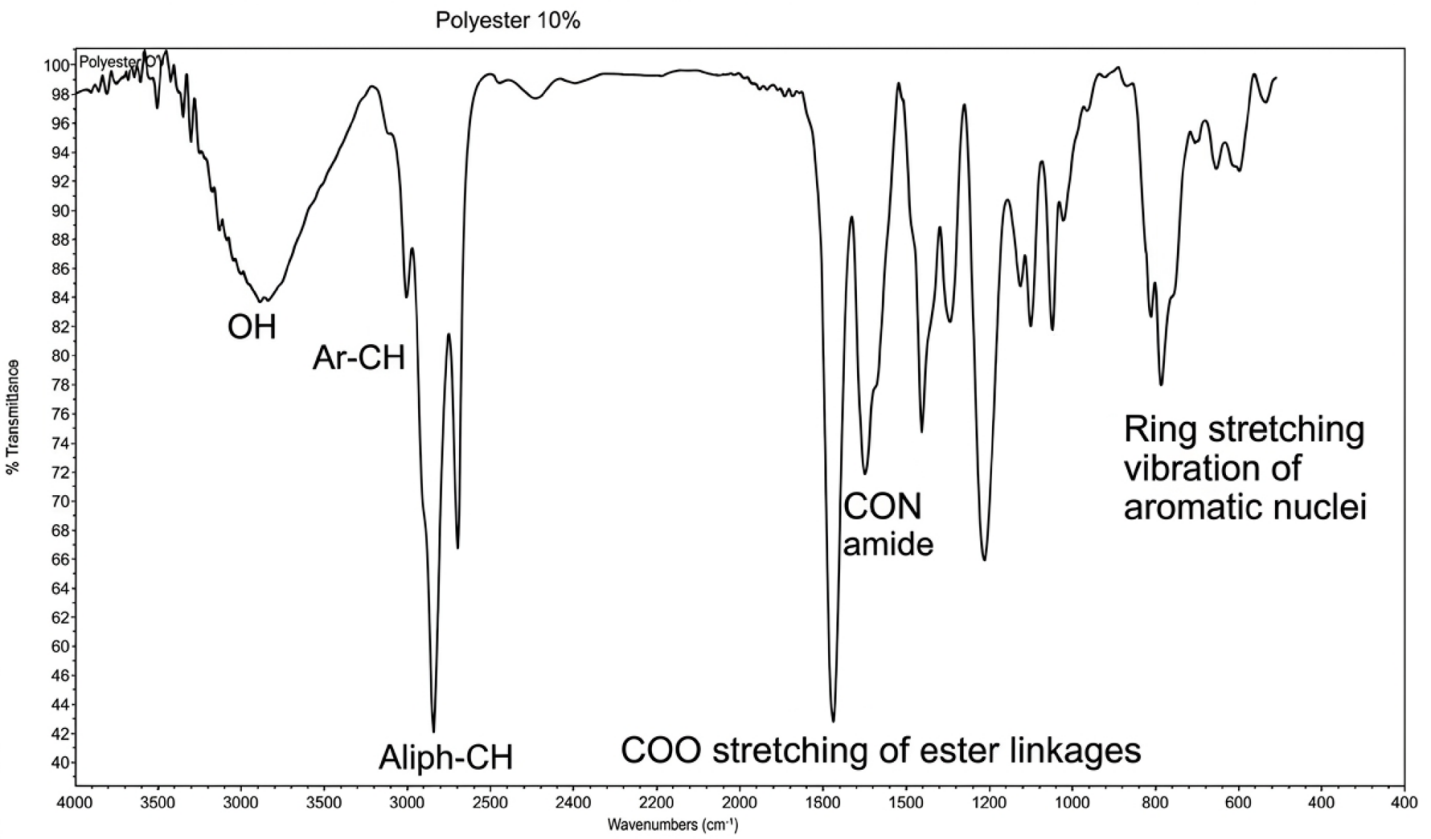
FT-IR spectra of the prepared modified PEA.

^1^***HNMR*** **(**Fig. 4**)** ***(δ***,*** ppm)*** = As shown in Fig. 4, the resonance signal for The signals of the methylene groups adjacent to the amide carbonyl (CO–CH2) are located at δ 2.14 ppm, whereas those attached to the amide nitrogen (N–CH_2_) appear at δ 3.02 ppm, confirming the formation of the amide linkage, and confirming the successful synthesis of the modified PEA resin through the incorporation of the new reactive modifier (HEHCCA), into the polymer backbone. Furthermore, the peaks at 7.03–7.76 ppm of the modified PEA correspond to the protons on the benzene ring skeleton. whereas signal at 0.82–0.84 ppm is due to the protons of the terminal methyl group. Furthermore, the signals of all protons of the internal CH2 groups present in the fatty acid chain appear at δ = 1.13–1.24 ppm. The signals at 5.28–5.32 ppm are assigned to the olefinic protons (–CH=CH–) of the fatty acid chains in alkyd and poly(ester-amide) resins^7^.

***IR (KBr)*** **(**Fig. 3**)**: ***ν***_***max***_ (cm^− 1^) = 3455 (OH), 3009 (arom. CH), 2930 (aliph. CH), 1740 (COO stretching of ester linkages), 1640 (CON amide carbonyl), 750 (Ring stretching vibration of aromatic nuclei)^9,30^.

**Fig. 4:**
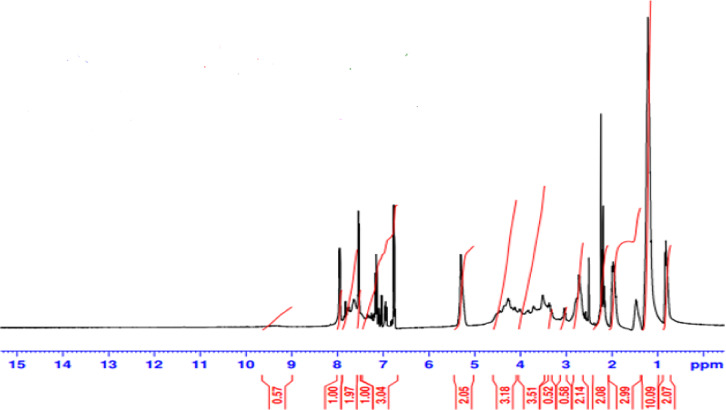
^1^H-NMR spectra of the prepared modified PEA.

### DSC&TGA interpretation of the prepared modified poly(ester-amide) resin

**Fig. 5:**
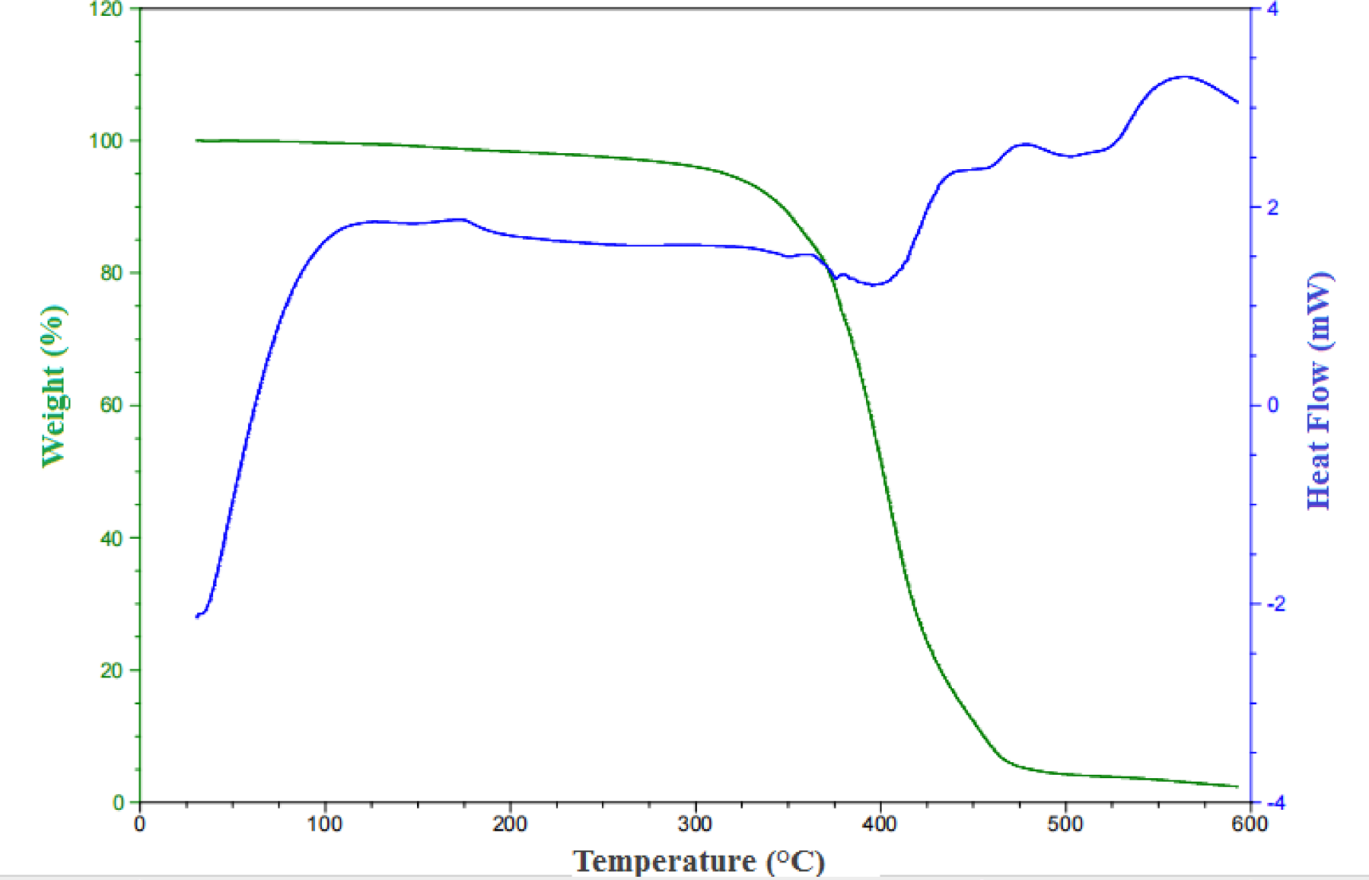
TGA and DSC curves of the prepared poly(ester-amide) (PEA).

The DSC/TGA thermogram of the prepared poly(ester-amide) (PEA) resin shows good thermal stability with a multistep degradation behavior characteristic of crosslinked polyester–amide systems (Fig. 5). From the TGA curve (green line), the resin exhibits very little weight loss below approximately 200 °C, indicating the absence of significant volatile impurities or residual solvent and confirming the good thermal integrity of the cured resin. The initial slight mass loss observed up to nearly 300 °C may be attributed to the evaporation of absorbed moisture, low-molecular-weight oligomers, or residual unreacted components. The main thermal degradation stage begins around 350–380 °C and proceeds rapidly up to nearly 470 °C. In this region, the resin undergoes major chain scission and decomposition of the ester and amide linkages present in the polymer backbone. The sharp decrease in weight% indicates the breakdown of the crosslinked polymeric network and volatilization of decomposition products such as CO, CO₂, hydrocarbons, and nitrogen-containing fragments. The maximum degradation rate appears near approximately 410–430 °C, suggesting that the prepared PEA resin possesses relatively high thermal resistance. Above nearly 480 °C, the degradation rate becomes slower and a small carbonaceous residue remains until 600 °C. The residual char yield is about 2–4%, which may be related to the formation of stable aromatic or carbonized structures during thermal decomposition. The low residual mass indicates that most organic constituents are volatilized during heating. The DSC curve (blue line) also supports the thermal behavior of the resin. A broad endothermic region at lower temperatures may correspond to physical relaxation and removal of entrapped moisture or volatile species. The absence of a sharp melting transition suggests that the prepared resin possesses an amorphous or highly crosslinked structure rather than a crystalline morphology. Around the major decomposition region (approximately 380–450 °C), noticeable thermal transitions appear due to bond cleavage and degradation reactions occurring simultaneously with mass loss in the TGA curve. These findings indicate that the prepared resin is thermally stable enough for coating and protective applications requiring moderate-to-high thermal resistance. The obtained DSC/TGA results are in good agreement with previously reported thermal behaviors of poly(ester-amide) resins and related thermosetting polyester–amide systems. In earlier studies, PEA materials generally exhibited initial thermal stability up to nearly 250–320 °C, followed by the principal degradation stage within the range of 350–500 °C due to cleavage of ester and amide bonds in the polymer backbone. Overall, the thermal analysis confirms that the synthesized modified PEA resin possesses thermal characteristics comparable to or better than many previously reported PEA coating materials, supporting its suitability for protective coating applications requiring enhanced thermal durability^4,7,34–36^.

### Physical characterization of the prepared modified PEA Resins

Based on the results summarized in Table 3, several important observations regarding the physical characteristics of the prepared HEHCCA-modified PEA resins can be made. First, the drying behavior of the prepared coatings was significantly influenced by the HEHCCA content. The drying time increased progressively with increasing modifier concentration. Samples A and B, containing 0% and 10% HEHCCA substitution, exhibited shorter drying times compared with samples containing 20% and 30% HEHCCA. Nevertheless, all coated samples reached complete hard-dry conditions after curing at 140 °C for 2 h. The increase in drying time may be attributed to the partial replacement of linseed fatty acid chains, which are rich in unsaturated drying sites, with the relatively rigid coumarin-containing HEHCCA structure. The reduction in unsaturation decreases the rate of oxidative cross-linking during curing, thereby prolonging the drying process. Second, the viscosity of the prepared resins increased slightly with increasing HEHCCA content at constant solid content. This behavior is likely associated with the higher molecular weight and increased intermolecular interactions introduced by the HEHCCA modifier. The presence of aromatic coumarin rings and amide functionalities enhances intermolecular hydrogen bonding and chain entanglement, leading to higher resistance to flow and consequently increased viscosity. Third, the prepared varnishes exhibited a dark brownish-yellow coloration with Gardner color values higher than 16. The increase in color intensity may be attributed to the incorporation of nitrogen-containing amide groups and the aromatic coumarin moiety within the modified PEA structure, which can contribute to enhanced chromophoric characteristics and slight thermal coloration during resin synthesis. Finally, the reaction time required for resin preparation increased with increasing HEHCCA percentage. This may be explained by the higher molecular weight and steric hindrance associated with the HEHCCA modifier, which reduce the mobility and reactivity of the reacting species during polycondensation. In addition, the aromatic structure of the coumarin derivative may decrease the overall reaction rate, resulting in longer synthesis times for highly modified resins^7^.

**Table 3:** Reaction time, Viscosity, Color, and Drying time Characteristics of various Modified PEA Resins.

Sample ID	Reaction time (minutes)	Acid value	Viscosity (mPa.s) at solid content = 70%	Color (Gardner)	Air drying (HD Time) (hr.)	Stoving dry (HD Time at 120 °C for (1 h.)	Stoving dry (HD Time at 140 °C for (3 h.)
A(0%)	55	10	60	16	7	HD	HD
B(10)	65	9	75	> 18	9	VST	HD
C(20)	80	10	85	> 18	15	ST	HD
D(30)	100	8	100	> 18	24	ST	HD

### Mechanical properties of dried films

**Fig. 6:**
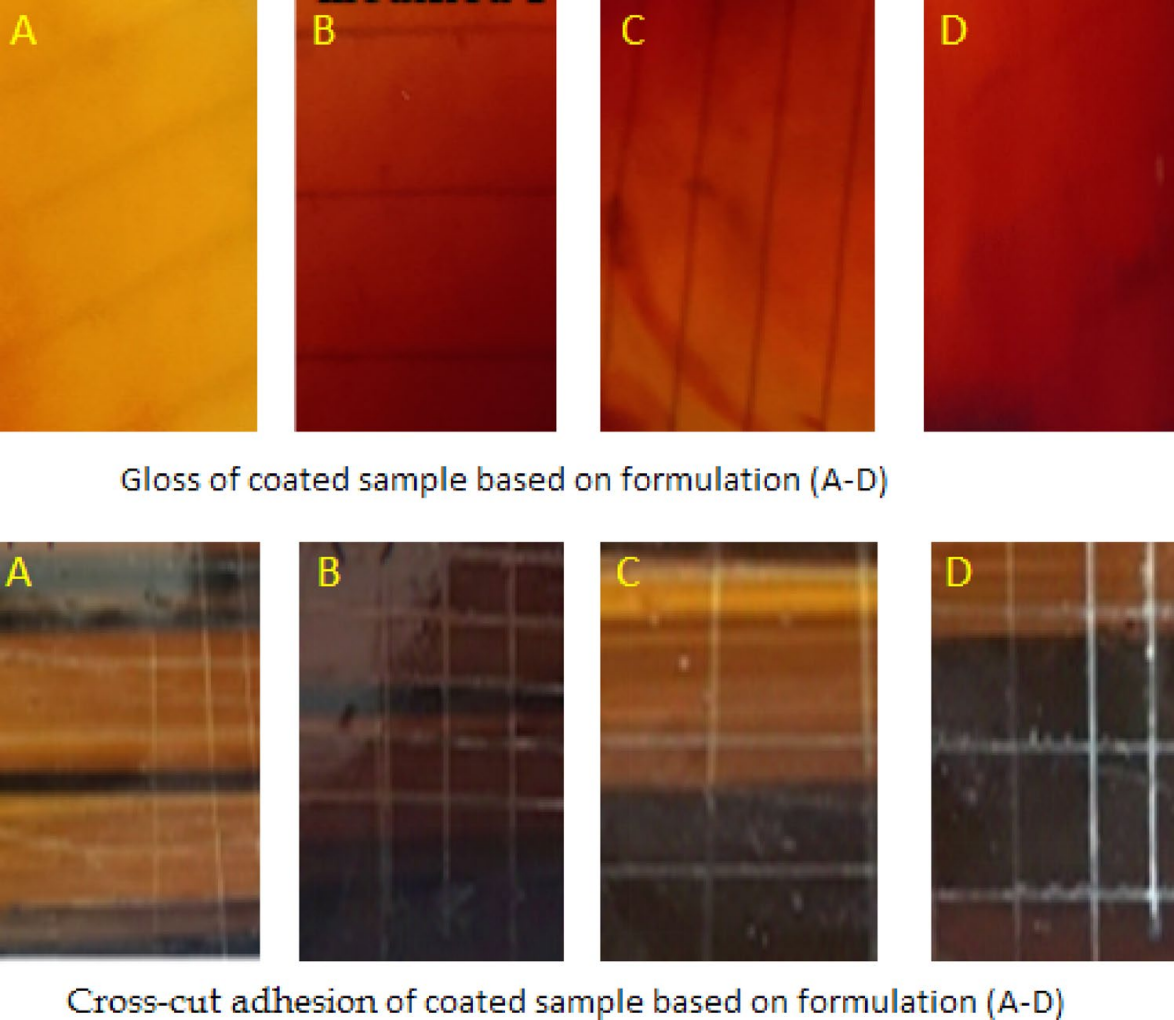
Photographic images of some mechanical characteristics for prepared of unmodified and modified PEA resins.

According to Table 4; Fig. 6, the mechanical properties of the modified PEA coatings were generally comparable; however, the HEHCCA-modified PEA coatings exhibited superior performance in several key parameters, including pencil hardness (2 kg vs. 1.5 kg), adhesion (5B vs. 4B), gloss (68 vs. 50), and impact resistance (1.5 J vs. 1.2 J). These enhancements can be attributed to the intrinsic structural characteristics of the poly(ester-amide) network and the incorporation of the HEHCCA modifier. The observed improvements in pencil hardness, adhesion, gloss, and impact resistance with the chemical structure of the modified PEA resin and the incorporation of the HEHCCA moiety are attributed to the presence of amide and ester functionalities is suggested to enhance intermolecular interactions and cohesive strength within the polymer matrix, while the aromatic coumarin structure may contribute to increased rigidity and reduced chain mobility. The presence of amide functionalities may promote stronger intermolecular interactions within the polymer matrix, which could contribute to reduced chain mobility and improved film hardness. Although direct crosslink density measurements were not performed, the enhanced mechanical properties suggest improved network integrity after HEHCCA incorporation. The increased polarity arising from amide and hydroxyl functionalities may improve interactions between the coating matrix and metallic substrate, contributing to the observed enhancement in adhesion. Overall, the incorporation of HEHCCA into the PEA resin system improved the balance between hardness, adhesion, flexibility, and impact resistance, indicating the beneficial role of the coumarin-based modifier in enhancing the performance of antimicrobial PEA coating formulations^7,15^.

**Table 4:** Mechanical characteristics and chemical resistance of various modified poly (ester- amide) resins.

Sample ID	DFT (µm)	Impact (J)	Gloss	Scratch hardness (kg)	Flexibility	Adhesion	Water resistance	Solvent resistance	Alkali resistance	Acid resistance
A(0%)	50 ± 5	1.2	50	1.5	Pass	4B	Pass	Pass	Poor	Pass
B(10)	50 ± 5	1.4	55	2	Pass	5B	Pass	Pass	Poor	Pass
C(20)	50 ± 5	1.4	60	> 2	Pass	5B	Pass	Pass	poor	Pass
D(30)	50 ± 5	1.5	68	> 2	Pass	5B	Pass	Pass	Poor	Pass

**Fig. 7:**
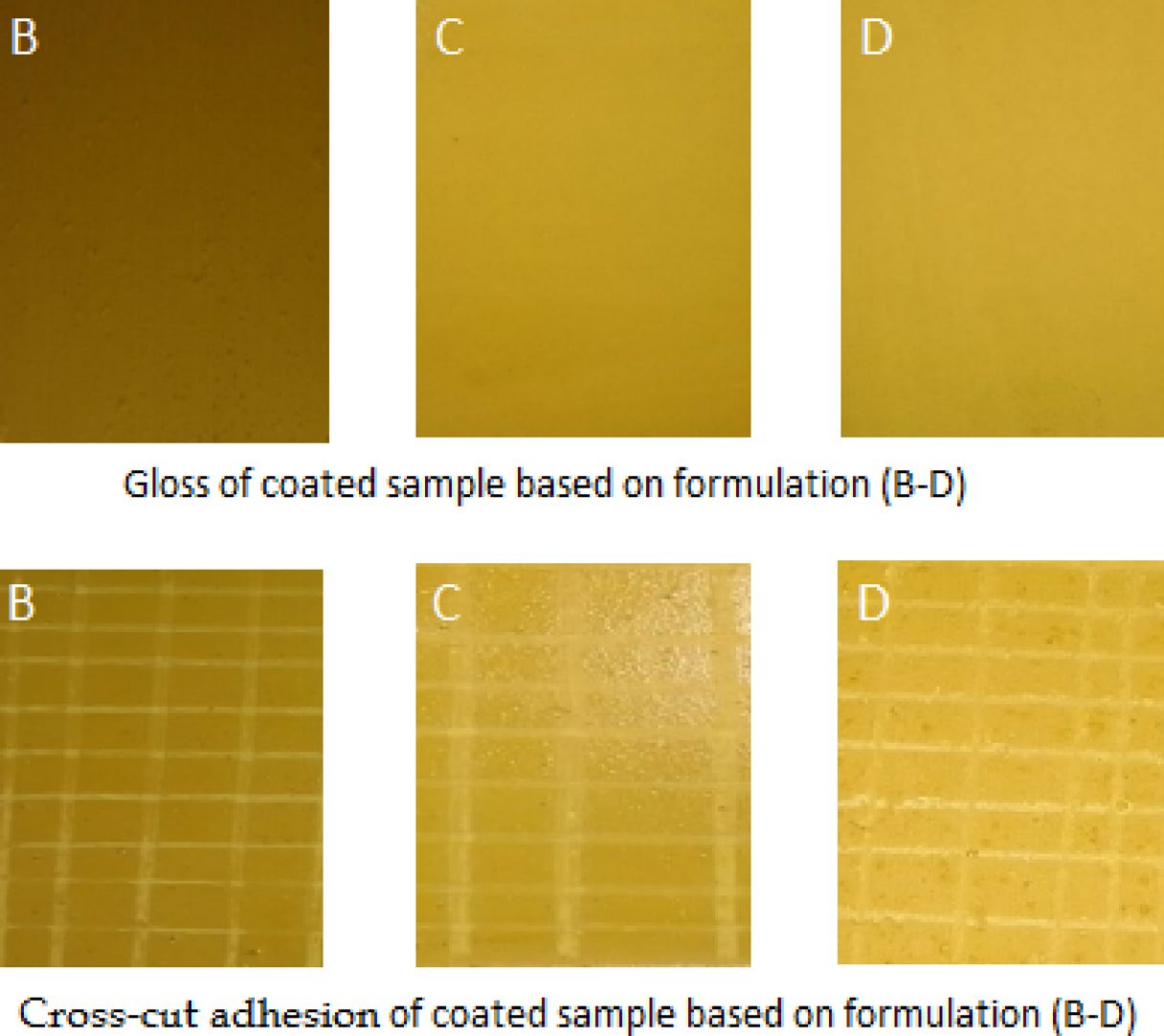
Photographic images of some mechanical characteristics for prepared paint based on modified PEA resins.

Chemical resistance measurements revealed excellent resistance toward water, acidic media, and organic solvents, indicating that incorporation of HEHCCA did not adversely affect coating stability under the employed experimental conditions. The enhanced resistance can be correlated with the compact cross-linked polymer network, which limits penetration of solvent molecules into the coating matrix. However, relatively poor alkali resistance was observed for all examined samples. This behavior can be explained by the susceptibility of ester groups within the PEA backbone to alkaline hydrolysis in the presence of sodium carbonate solution. Under alkaline conditions, cleavage of ester linkages occurs through nucleophilic attack of hydroxide ions, leading to partial degradation of the polymer network and deterioration of film properties. Although the increased cross-link density introduced by HEHCCA improves the compactness of the coating matrix, it cannot completely suppress hydrolytic attack in strongly alkaline environments. Therefore, alkali resistance remains the principal limitation of these modified PEA systems. Regarding the final pigmented paint formulations, no significant changes were observed in most mechanical properties after pigment incorporation, except for impact resistance and scratch hardness, which increased progressively with increasing HEHCCA content, as presented in Table 5; Fig. 7. This behavior further supports the role of HEHCCA in increasing cross-link density and reinforcing the polymeric coating network.

**Table 5:** Mechanical characteristic of the painted films based on modified poly (ester- amide) resins.

Sample ID	DFT (µm)	Impact (J)	Gloss	Scratch hardness (kg)	Flexibility	Adhesion
A(0%)	100 ± 5	1.4	55	1.5	Pass	5B
B(10)	100 ± 5	1.6	60	2	Pass	5B
C(20)	100 ± 5	1.8	70	> 2	Pass	5B
D(30)	100 ± 5	1.9	70	> 2	Pass	5B

### Antimicrobial activity of various modified poly(ester-amide) resins

**Fig. 8:**
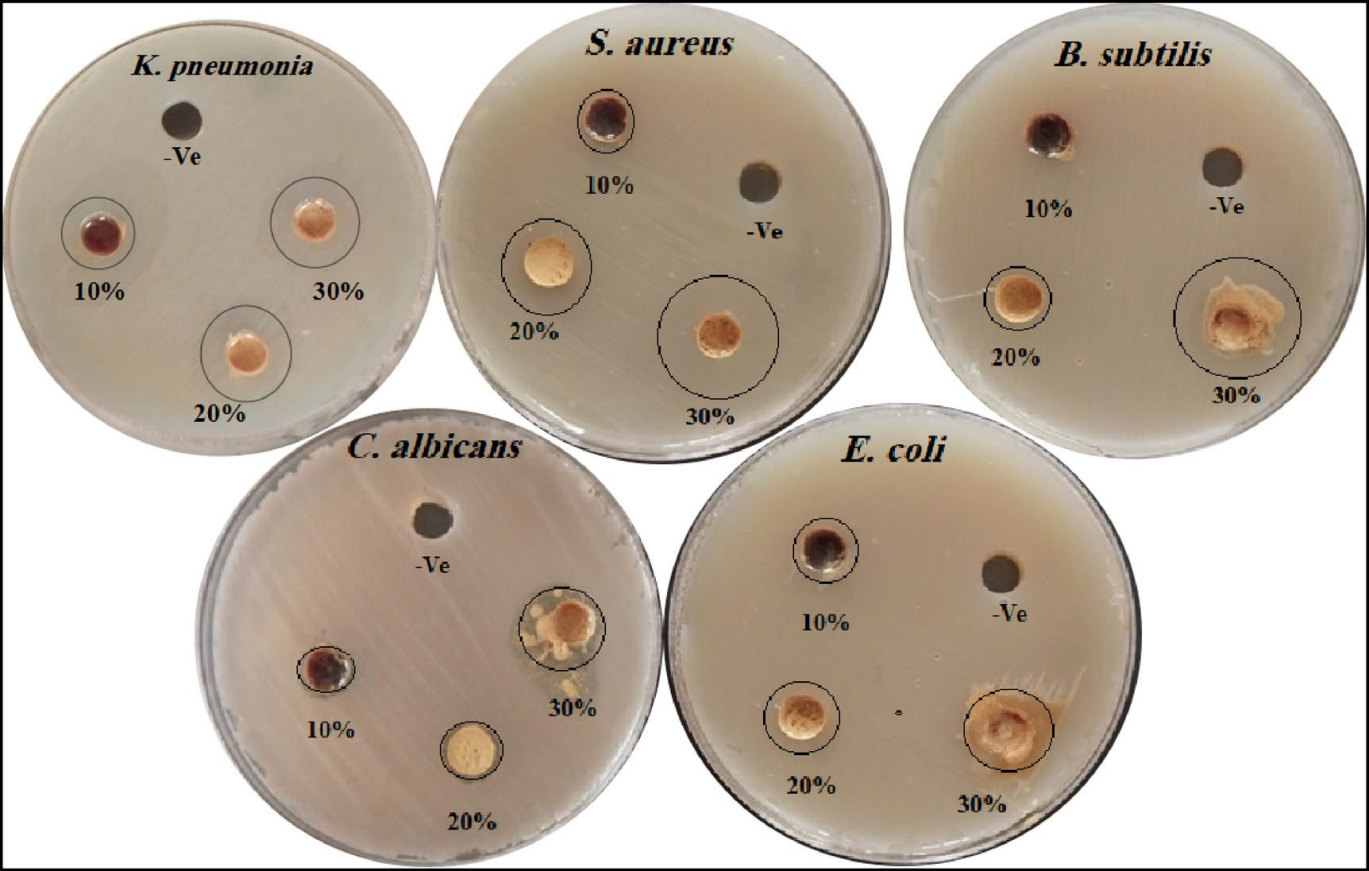
Antimicrobial activity of the paint formulation based on unmodified PEA resin and 10, 20 and 30% HEHCCA modified PEA resins. Unmodified PEA (blank) as a negative control (−Ve) toward *E. coli*, *K. pneumonia*, *B. subtilis*, *S. aureus*, and *C. albicans*.

**Fig. 9:**
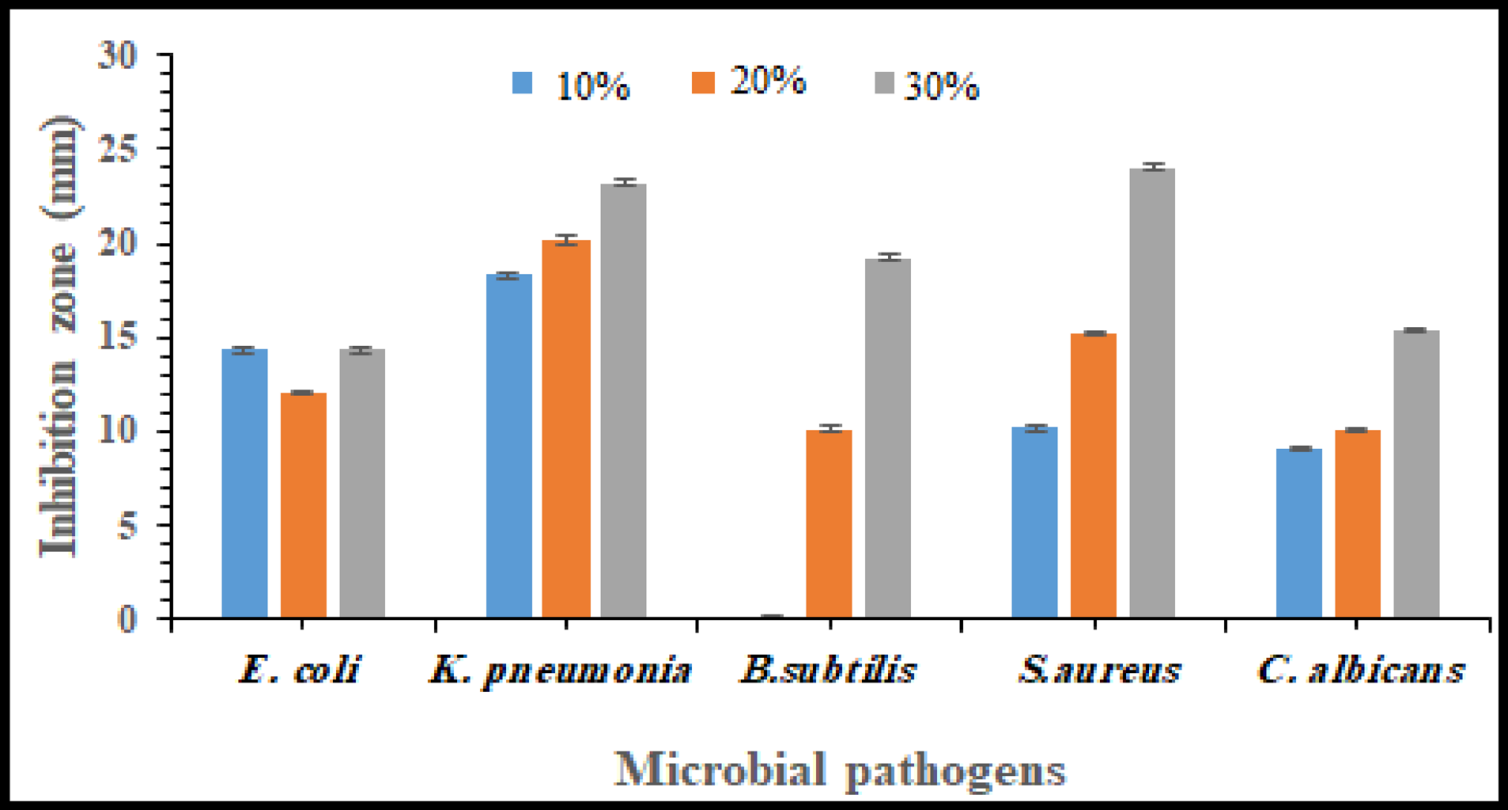
Inhibition zones of the prepared paint formulations based on HEHCCA-modified poly(ester-amide) resins against various bacterial and fungal strains.

The antimicrobial activity of the prepared paint formulations based on HEHCCA-modified PEA resins was evaluated against selected Gram-positive bacteria, Gram-negative bacteria, and unicellular fungi using the agar-well diffusion method, as illustrated in Figs. 8 and 9. The results demonstrated that the modified coatings exhibited broad-spectrum antimicrobial activity, with effectiveness strongly dependent on the HEHCCA content within the polymer backbone. Among the tested samples, the paint formulation containing 30% HEHCCA-modified PEA resin exhibited the highest antimicrobial performance. *Staphylococcus aureus* showed the greatest susceptibility, with an inhibition zone of 24.15 ± 0.30 mm. The same formulation also displayed significant inhibitory activity against *Klebsiella pneumoniae* (23.20 ± 0.15 mm), *Bacillus subtilis* (19.20 ± 0.26 mm), *Candida albicans* (15.30 ± 0.21 mm), and *Escherichia coli* (14.30 ± 0.30 mm). For the coating based on 20% HEHCC-modified PEA resin, *K. pneumoniae* remained the most sensitive strain with an inhibition zone of 20.20 ± 0.20 mm, whereas *S. aureus*, *E. coli*, *B. subtilis*, and *C. albicans* exhibited comparatively lower inhibition values. Similarly, the formulation containing 10% HEHCCA-modified PEA resin showed weaker antimicrobial activity overall, with *B. subtilis* exhibiting complete resistance under the tested conditions. The observed increase in antimicrobial activity with increasing HEHCCA content indicates a clear structure–activity relationship between the concentration of the coumarin-based modifier and biological performance. This enhancement may be attributed to several synergistic factors. First, the coumarin nucleus is well known for its intrinsic antimicrobial properties due to its ability to interfere with microbial enzymatic systems and membrane functions. Second, the phenolic hydroxyl groups present in the coumarin structure can promote oxidative stress and disruption of microbial cell membranes through hydrogen-bond interactions with cellular proteins. The antimicrobial selectivity observed between Gram-positive and Gram-negative bacteria can also be explained by differences in cell wall structure. Gram-positive bacteria such as *S. aureus* possess a relatively porous peptidoglycan layer without an outer lipopolysaccharide membrane, allowing easier penetration of active coumarin-containing species into the bacterial cell. In contrast, Gram-negative bacteria such as *E. coli* possess an additional outer membrane rich in lipopolysaccharides that acts as a permeability barrier, thereby reducing susceptibility toward antimicrobial agents. This explains the comparatively lower inhibition zones observed for *E. coli*^37,38^. Overall, the obtained results demonstrate that HEHCCA-modified PEA resins, particularly at 30% modification level, provide a promising platform for development of multifunctional antimicrobial coatings combining enhanced mechanical durability with effective antimicrobial performance. So, in future work we will investigate the relationship between mechanical durability and retained antimicrobial efficiency after prolonged abrasion exposure.

## Conclusion

In this study, novel antimicrobial coating formulations based on modified poly(ester-amide) (PEA) resins were successfully developed using 3-*N*-bis(2-hydroxyethyl)-7-hydroxy coumarin carboxamide (HEHCCA) as a reactive modifier. The modification strategy involved the partial replacement of hydroxyethyl linseed fatty acid amide (HELA) with HEHCCA as a polyhydric alcohol source during resin synthesis. This approach represents a new route for introducing coumarin-based bioactive functionalities directly into the PEA backbone, leading to multifunctional coating materials with enhanced performance characteristics. The synthesized modified PEA resins were successfully prepared and structurally confirmed through the characteristic functional groups identified in accordance with our previous investigations. The obtained resins were further utilized as binders in paint formulations and evaluated for their physico-mechanical and antimicrobial properties. The incorporation of HEHCCA significantly improved the coating performance compared with the unmodified PEA system and commercial reference coatings. The modified formulations exhibited higher gloss values (68 vs. 50), superior hardness (> 2 kg vs. 1.5 kg), improved adhesion (5B vs. 4B), and enhanced impact resistance (> 1.5 J vs. 1.2 J), demonstrating the positive effect of the coumarin-containing modifier on coating durability and film integrity. Biological evaluation revealed that the antimicrobial activity increased progressively with increasing HEHCCA content in the polymer backbone, following the order F1 < F2 < F3 < F4. Among all formulations, the 30 wt% HEHCCA-modified PEA coating exhibited the highest antimicrobial efficiency in both wet and dry coated films. The enhanced antimicrobial performance may be attributed to the synergistic effect of the coumarin nucleus, phenolic hydroxyl groups, and amide linkages, which collectively contribute to improved microbial inhibition, intermolecular hydrogen bonding, and increased crosslinking density within the coating matrix. Overall, the present work demonstrates that modified PEA resins can serve as promising multifunctional antimicrobial binders with simultaneous improvements in mechanical durability and biological resistance. The developed coatings show strong potential for advanced industrial applications, particularly in hygienic, protective, and antimicrobial surface coatings. Further studies involving long-term durability, large-scale production, and field performance evaluation are recommended to facilitate practical industrial implementation.

## Data Availability

The data used to support the findings of this study are available from the corresponding author upon request.
